# Synergistic effects of *Piper ornatum* and lemuru fish oil on *in vitro* rumen fermentation, nutrient digestibility, and methane mitigation

**DOI:** 10.14202/vetworld.2026.1914-1931

**Published:** 2026-05-12

**Authors:** Mardiati Zain, Despal Despal, Ujang Hidayat Tanuwiria, Yunilas Yunilas, Roni Pazla, Ezi Masdia Putri, Gusri Yanti, Zaitul Ikhlas, Rifa Ratna Sari, Laras Sukma Sucitra, Bella Veliana Utami

**Affiliations:** 1 Department of Animal Nutrition and Feed Technology, Faculty of Animal Husbandry, Universitas Andalas, Padang, West Sumatra, Indonesia; 2 Department of Animal Nutrition and Feed Technology, Faculty of Animal Science, IPB University, Bogor, West Java, Indonesia; 3 Ruminant and Feed Chemistry Laboratory, Department of Animal Nutrition and Feed Technology, Faculty of Animal Sciences, Universitas Padjadjaran, Sumedang, West Java, Indonesia; 4 Department of Animal Science, Faculty of Agriculture, Universitas Sumatera Utara, Medan, Indonesia; 5 Research Center for Animal Husbandry, National Research and Innovation Agency (BRIN), Cibinong, Indonesia; 6 Faculty of Social Science and Education, Prima Nusantara Bukittinggi University, West Sumatra, Indonesia; 7 Graduate Program, Faculty of Animal Science, Universitas Andalas, Padang, West Sumatra, Indonesia

**Keywords:** essential oils, methane mitigation, nutrient digestibility, *Piper ornatum*, polyunsaturated fatty acids, rumen fermentation, sustainable livestock, *in vitro*

## Abstract

**Background and Aim:**

Enteric methane emissions from ruminants represent a major loss of dietary energy and contribute significantly to greenhouse gas accumulation. Phytogenic feed additives and lipid supplements have emerged as sustainable strategies to modulate rumen fermentation and mitigate methanogenesis. This study evaluated the synergistic effects of Piper ornatum (PO) leaf powder and lemuru fish oil (LFO) on in vitro rumen fermentation characteristics, nutrient digestibility, and methane reduction.

**Materials and Methods:**

A 4 × 4 factorial randomized block design was used, with four levels of PO (0%, 3%, 6%, and 9%) and LFO (0%, 1.5%, 3%, and 4.5%), and three replications. The basal diet was incubated with buffered rumen fluid for 48 h at 39°C. Parameters measured included microbial protein synthesis (MPS), protozoa population, partial and total volatile fatty acids (VFAs), ruminal pH, ammonia (NH_3_) concentration, total gas production, methane production, and nutrient digestibility (dry matter, organic matter, crude protein, and fiber fractions).

**Results:**

The combined supplementation significantly influenced rumen fermentation (p < 0.05). Methane production and total gas output decreased markedly with increasing additive levels, with the lowest methane value (7.46 mL/g DM) observed at 6% PO and 4.5% LFO. Protozoa populations declined, indicating antimethanogenic effects. In contrast, MPS (up to 405.47 mg/100 mL), total VFA concentration (up to 123.51 mM), and NH_3_ production increased significantly, reflecting enhanced microbial activity and nitrogen utilization. Nutrient digestibility, including dry matter, organic matter, crude protein, and fiber fractions, improved significantly across treatments. Ruminal pH remained stable (6.90–6.99), indicating no disruption of the fermentation environment.

**Conclusion:**

The combination of 6% PO and 4.5% LFO provided the most favorable balance between methane mitigation, fermentation efficiency, and nutrient utilization. This synergistic strategy integrates phytogenic bioactive compounds and polyunsaturated fatty acid-mediated hydrogen diversion, offering a promising approach for sustainable ruminant production. However, in vivo validation is required to confirm long-term effectiveness under practical feeding conditions.

## INTRODUCTION

Methane emissions from ruminants represent both a loss of dietary energy and a major contributor to greenhouse gases, thereby reducing livestock production efficiency and negatively affecting meat and milk productivity. Therefore, methane mitigation is essential for enhancing feed efficiency and ensuring the sustainability of the livestock industry. Efforts to reduce methane emissions can improve the efficiency of feed energy utilization in ruminants. Various feed additives have been investigated; however, their effectiveness has not been consistently confirmed under in vivo conditions. Potential feed additives include plants containing bioactive compounds, such as flavonoids and essential oils. Flavonoids and essential oils are plant secondary metabolites with antimicrobial properties that act similarly to antibiotics as either bacteriostatic (inhibiting bacterial growth) or bactericidal (killing bacteria) [1, 2]. Their antibiotic-like properties, particularly against methanogenic microorganisms such as archaea and protozoa, contribute to the reduction of methane production [1, 3].

Many plants in Indonesia contain secondary metabolites (such as flavonoids, essential oils, saponins, and tannins) that can be utilized as feed additives to reduce methane production; however, their effects have not yet been fully confirmed under in vivo conditions. These include red betel (*Piper ornatum [PO]*) leaves, pineapple peel, dragon fruit peel, key lime peel, guava leaves, acacia, and mangrove plants, all of which possess bioactive compounds capable of reducing ruminal biohydrogenation and lowering methane emissions. *Piper betle* leaves contain flavonoids and essential oils (including catechin, rutin, apigenin, myricetin, kaempferol, and caryophyllene), which have the potential to alter fermentation pathways related to pyruvate metabolism and ruminal biohydrogenation. *P. betle* is rich in flavonoids and phenolic compounds with established antimicrobial properties. Its active compounds modulate rumen microbes and improve rumen fermentation [4, 5]. The inclusion of *P. betle* at 15–30 mg *in vitro* reduced methane production while improving rumen fermentation [6]. In addition, saponins have the ability to improve feed digestibility; therefore, they increase the total concentration of volatile fatty acids (VFA) (acetic acid, propionic acid, and butyric acid), which serve as important energy sources for livestock [7, 8].

Lemuru fish oil (LFO), rich in polyunsaturated fatty acids (PUFAs) such as eicosapentaenoic acid and docosahexaenoic acid, has been reported to serve as a significant hydrogen sink in the rumen. The incorporation of PUFA into the diet provides an alternative pathway for hydrogen utilization, thereby limiting the availability of free hydrogen for methanogenic archaea and reducing methane production. Progressive supplementation of LFO up to 7% of the total ration reduced enteric methane emissions by approximately 17–31% without adversely affecting rumen fermentation [9]. Beyond its role in methane mitigation, PUFA supplementation is associated with improved rumen fermentation efficiency, potential shifts in microbial populations, and enhanced beneficial fatty acid profiles in ruminant-derived products. These properties highlight LFO as a promising feed additive for improving both environmental sustainability and the nutritional quality of ruminant production systems.

LFO contains omega-3 fatty acids derived from the canning process, accounting for 29.68% of its total fatty acid content. In addition, fish oil rich in omega-3 fatty acids exhibits antioxidant properties [10]. LFO is also reported to contain PUFA, representing 75% of the total fatty acids [11]. The high PUFA and conjugated linoleic acid content in LFO make it a promising feed additive for ruminants. Supplementation with PUFA has been shown to improve energy efficiency by increasing dietary energy density and enhancing tissue protein synthesis through increased non-ammonia nitrogen flow to the duodenum [12].

Despite extensive research on phytogenic additives and lipid supplementation independently, there remains a significant knowledge gap regarding their combined effects on rumen fermentation dynamics, nutrient utilization, and methane mitigation. Most previous studies have evaluated plant-derived bioactive compounds or PUFA-rich lipid sources separately, with limited integration of both strategies into a unified approach. Furthermore, although several plant species have demonstrated antimethanogenic potential, *PO* remains underexplored in ruminant nutrition compared to other Piper species such as *P. betle*. Similarly, while LFO supplementation has been widely studied, the synergistic interaction between phytogenic compounds and lipid-derived hydrogen sinks has not been adequately investigated under *in vitro* conditions.

In addition, the mechanistic understanding of how combined phytogenic–lipid supplementation influences microbial protein synthesis (MPS), protozoal populations, and fermentation end-products remains insufficient. Previous findings have shown variability in fermentation responses across dosage, substrate composition, and microbial adaptation, highlighting the need for systematic evaluation with controlled experimental designs. Moreover, there is limited evidence regarding the optimal inclusion levels of such combined additives that can simultaneously enhance fermentation efficiency while minimizing methane production without compromising ruminal stability.

Therefore, this study was designed to evaluate the synergistic effects of *PO* leaf powder and LFO supplementation on rumen fermentation characteristics, nutrient digestibility, and methane production under *in vitro* conditions. Specifically, the study aimed to determine the optimal inclusion levels of both additives that maximize microbial protein synthesis, improve VFA production, and reduce methane emissions while maintaining stable ruminal pH. In addition, this study sought to provide mechanistic insights into how the combination of phytogenic bioactive compounds and PUFA-mediated hydrogen diversion can enhance fermentation efficiency and nutrient utilization. The findings are expected to contribute to the development of sustainable feeding strategies for ruminant production systems, particularly by using locally available feed resources that serve dual functions: improving productivity and mitigating environmental impact.

## MATERIALS AND METHODS

### Ethical approval

This study used *in vitro* rumen fermentation techniques and did not involve live animal experimentation during the experimental phase. Rumen fluid used for the *in vitro* incubation was obtained from donor ruminants maintained under standard management conditions. The collection of rumen fluid was performed in accordance with established animal welfare guidelines and standard operating procedures to minimize animal stress and discomfort.

All procedures related to the handling and sampling of donor animals were carried out in accordance with institutional and national guidelines for the care and use of animals in research, based on the Republic of Indonesia government law 18 of 2009 (Section 66). The protocol for rumen fluid collection was reviewed and approved by Indonesia Nasional Standard 99003:2018 with registration No. 170/170/KEP/BSN/7/2018.

The study complied with internationally accepted ethical standards for animal use in research and adhered to relevant guidelines for *in vitro* rumen fermentation studies.

### Study period and location

This study was conducted from May to October 2025 at the Ruminant Laboratory of the Faculty of Animal Science, Andalas University.

### Substrate preparation

The basal substrate used for *in vitro* fermentation consisted of a 50% roughage: 50% concentrate ratio, formulated to represent a typical ruminant diet. The roughage component was indigofera, elephant grass, and gamal, collected from experimental garden of Animal Husbandry Faculty, Andalas University, while the concentrate consisted of corn, palm kernel meal, sago, rice bran, and minerals.

### Additive preparation and characterization

Leaves of *PO* were shade-dried at ambient temperature (25–30°C) for 48–72 h, then oven-dried at 40–45°C until a constant weight was achieved, to minimize loss of volatile bioactive compounds. The dried material was ground to pass a 1-mm sieve and stored in airtight, light-protected containers at 4°C prior to use. The powder was then incorporated into the substrate at 0%, 3%, 6%, and 9% DM. The bioactive compounds in *PO* leaf powder are shown in Table 1.

**Table 1:** Bioactive compounds in *Piper ornatum* detected through metabolomic analysis.

Group	Compound
Fatty acids and derivatives	9-Oxo-ODE
	α-Eleostearic acid
	(+/−)9,10-dihydroxy-12Z-octadecenoic acid
	Ethyl palmitoleate
	L-α-PALMITIN
	Erucamide
	Stearamide
Terpenoid (monoterpene, sesquiterpene, triterpene, carotenoid)	Curcumene
	(E, E)-α-Farnesene
	(−)-Caryophyllene oxide
	Nootkatone
	Echinenone
	Gomisin H
Phenolics and aromatic phenylpropanoid	3,5-Dihydroxyphenylpropionic acid
	Cinnamaldehyde
	Coumarin
	(E)-4-Methoxycinnamic acid
	Phloroacetophenone
	Zingerol
	Benzoic acid
	3,4-Dihydroxybenzaldehyde
	Naphthalene-2,3-diol
Organic acids and energy metabolism-related metabolites	Citric acid
	Gluconic acid
	L-Pyroglutamic acid
Amino acids and derivatives	D-(+)-Pipecolinic acid
	DL-Stachydrine
	Betaine
	1-[(3-Carboxypropyl)amino]-1-deoxy-β-D-fructofuranose
Alkaloids and nitrogen-containing compounds	Cinchonidine
	6-Acetylcodeine
	Imagabalin
	Urea, N,N’’-1,2-ethenediylbis(N-nitroso-
Sterols and derivatives	Sitostenone
	(3β,24R,24′R)-fucosterol epoxide
	2-Methoxyestrone
Aromatic hydrocarbons and benzene derivatives	Indane (allylbenzene)
	Cumene
	3-Methyl-1-phenyl-2-butene
	1-Thiochromone
	Phthaldialdehyde
Complex esters, amides, and synthetic compounds	Bis(4-ethylbenzylidene)sorbitol
	Kresoxim-methyl
	4,4-Bis[4-(acetyloxy)phenyl]3-hexanone
	2-(3-Cyano-3-((3-phenylpropoxy)karbonil)-2-propenilidena)-1,3,3-trimetil-2,3-dihydro-1H-indole-5-karboksilat, metil ester
	Methyl isonicotinate
	(2E)-3-(4-Hydroxyphenyl)-N-[2-(4-hydroxyphenyl)ethyl]acrylamide
	Bis(4-ethylbenzylidene)sorbitol
Pigments and chlorophyll derivatives	Pheophorbide A
Quaternary ammonium compound	Choline
Synthetic phenolic antioxidants	3-BHA

3-BHA = butylated hydroxyanisole.

Meanwhile, LFO was obtained from a commercial fish oil producer, stored at 4°C in the dark to prevent oxidation, and added at 0%, 1.5%, 3%, and 4.5% DM. The major fatty acid profile of LFO is shown in Table 2.

**Table 2:** Fatty acid profiles of lemuru fish oil.

Classification	Fatty acid	Concentration (μg/mL)
SFA	Hexanoic acid	0.00
SFA	Octanoic acid	0.00
SFA	Decanoic acid	0.00
SFA	Undecanoic acid	0.00
SFA	Dodecanoic acid	1.61
SFA	Tridecanoic acid	0.77
SFA	Myristic acid	195.15
SFA	Pentadecanoic acid	17.72
SFA	Hexadecanoic acid (palmitic acid)	690.20
SFA	Heptadecanoic acid	1.52
SFA	Octadecanoic acid (stearic acid)	193.37
SFA	Methyl icosanoate	13.85
SFA	Heneicosanoic acid	2.20
SFA	Tricosanoic acid	4.03
MUFA	Cis-9-tetradecenoic acid	0.72
MUFA	Cis-10-pentadecenoic acid	0.00
MUFA	9-Hexadecenoic acid (palmitoleic acid)	280.94
MUFA	Cis-10-heptadecenoic acid	1.52
MUFA	Methyl trans-9-elaidate	0.00
MUFA	9-Octadecenoic acid (oleic acid)	242.19
MUFA	Cis-13,16-docosadienoic acid	22.11
MUFA	13-Docosenoic acid (methyl erucate)	5.82
PUFA	Cis-6,9,12-octadecatrienoic acid	6.49
PUFA	9,12-Octadecadienoic acid (linoleic acid)	41.66
PUFA	9,12,15-Octadecatrienoic acid (α-linolenic acid)	9.85
PUFA	8,11,14-Eicosatrienoic acid	0.00
PUFA	5,8,11,14-Eicosatetraenoic acid (arachidonic acid)	55.11
PUFA	Methyl eicosa-5,8,11,14,17-pentaenoate (EPA)	265.92
PUFA	Methyl eicosa-5,8,11,14,17-pentaenoate	177.56
PUFA	9,12-Octadecadienoic acid	41.66

SFA = saturated fatty acids, MUFA = monounsaturated fatty acids, PUFA = polyunsaturated fatty acids, EPA = eicosapentaenoic acid.

Fatty acid profiles of LFO were further categorized based on the degree of saturation into saturated fatty acids, monounsaturated fatty acids, and PUFA, according to the number of double bonds present in their carbon chains.

### Experimental treatment

The objective of this experiment was to determine the optimal levels of red betel leaf (*PO*) and LFO supplementation to improve ration digestibility, optimize rumen fermentation products, and reduce methane production. The ration was formulated with four levels of each additive, namely *PO* at 0%, 3%, 6%, and 9%, and LFO at 0%, 1.5%, 3%, and 4.5%. The experimental design was a randomized block design with a 4 × 4 factorial arrangement and three replications. The three replications corresponded to rumen fluid collected from three different Kacang goats, with each goat serving as a block to account for inter-animal variation in rumen microbial composition and fermentation activity. Within each block, all treatment combinations were incubated, and each treatment was prepared in triplicate bottles as technical replicates. The mean value of the triplicate bottles within each block was used as the experimental unit for statistical analysis. The variables observed included ration fermentability and digestibility. Ration fermentability was assessed by measuring the digestibility of DM, organic matter, crude protein, and fiber fractions, and by producing total and partial VFA, including acetate, propionate, butyrate, valerate, and iso-acids. Protein fermentability was evaluated by measuring NH_3_ concentration, and rumen condition was measured through ruminal pH. Additional measurements included MPS, microbial populations (bacteria and protozoa), total gas production, and methane production. A metabolomic analysis to identify the bioactive compounds in *PO* was conducted at BRIN using liquid chromatography, high-resolution mass spectrometry, and untargeted metabolomics. The fatty acid profile of LFO was determined using a gas chromatograph 8700 fitted with a flame ionization detector, following Khan’s method [13].

### *In vitro* experiment

The *in vitro* test followed the method of Tilley and Terry [14]. The present study used rumen liquor collected from Kacang goats (four head) immediately after slaughter in the morning at an authorized municipal abattoir under the supervision of official veterinary officers, ensuring adherence to animal welfare and biosafety protocols. The animals were fed a diet consisting of elephant grass, legumes, and concentrate. Fresh rumen fluid was passed through a 100 μm nylon sieve and transferred into thermos flasks preheated to 39°C. The filtrate was diluted with McDougall’s buffer at a 1:4 ratio (rumen fluid: buffer) [15]. Four Erlenmeyer flasks were used per treatment. The pH value of the mixture was 7.2. For each incubation, 2.5 g of sample was added to 250 mL of the rumen fluid–buffer mixture in Erlenmeyer flasks. Anaerobic conditions were established by flushing the headspace with carbon dioxide, after which the flasks were sealed with rubber stoppers. The flasks were incubated in a continuous shaking incubator at 39°C and 90 rpm for 48 h. Fermentation was stopped by placing bottles in ice water, and the pH was measured post-incubation with a digital pH meter.

After incubation, the residue and supernatant were separated by centrifugation at 191 × *g* for 5 min at 4°C. The residue was used to determine nutrient digestibility. The digestibility of DM, organic matter, and crude protein was determined following the AOAC method [16]. Fiber fraction digestibility was determined following the Van Soest method [17]. Total VFA concentration was measured by steam distillation, whereas NH_3_ concentration was determined by Conway microdiffusion using 1 mL of supernatant [18]. Partial VFA analysis was conducted using gas chromatography fitted with a flame ionization detector. Separation was carried out using a stainless-steel column packed with GP 107, SP 1,000/L % H3 PO4 on Chromosorb WAW (100/120 mesh) [19]. MPS was measured using the Lowry method [20]. An aliquot of each sample was reacted with Lowry reagents A and B, and the absorbance was measured at 750 nm using a spectrophotometer. Protein concentration was subsequently calculated from the regression equation derived from an appropriate protein standard curve.

The protozoa population was assessed following Ogimoto and Imai by mixing 1.0 mL of supernatant from each sample with 1.0 mL of MF fixative solution to immobilize and preserve protozoa. The mixture was homogenized gently before counting. Two drops of the fixed filtrate were placed in a counting chamber and covered with a coverslip to ensure uniform distribution. The counting chamber had a depth of 0.1 mm and consisted of 16 grids, with the smallest grid area of 0.0625 mm²; only four designated grids were counted per chamber to obtain the mean count. Protozoa were enumerated under a light microscope using a 40× objective lens and 10× ocular lens (total magnification 400×). Protozoal concentration (cells/mL) was calculated using the following formula:

Protozoa population (cell/mL) = 1 × 100 × C × Fp / (0.1 × 0.0625 × 16 × 5),

where C is the average number of protozoa counted per grid, and Fp is the dilution factor arising from sample fixation and handling [21].

Gas production was measured using the Theodorou method at 48 h [22], and methane production was measured following Yanza et al. [23]. The blank incubation (inoculum and buffer only) produced 26.0 ± 1.41 mL of total gas per incubation at 48 h, with methane production of 5.0 ± 0.82 mL (n = 4). These values were used to correct all treatment measurements. All data obtained were subjected to analysis of variance and further tested using Duncan’s multiple range test. Data were analyzed using SPSS v25.0 software (IBM Corp., New York, USA). Bioactive compounds in *PO*, fatty acid profiles of LFO, nutrient content, and feed formulation are described in Tables 1–4.

**Table 3:** Nutritional content of basal and concentrate feed ingredients (%).

Nutrient content	Indigofera	Elephant grass	Gamal	Corn	Palm kernel meal	Sago	Rice bran
DM	92.84	93.39	91.86	85.55	85.91	93.60	85.87
OM	91.41	90.88	93.01	95.87	96.13	90.20	88.99
CP	30.92	9.66	21.79	9.82	22.29	2.56	8.94
Ash	9.59	2.06	6.99	3.09	10.99	9.80	8.76
CF	17.43	32.60	23.45	9.61	29.96	10.80	15.62
NFE	40.67	41.86	43.84	73.35	32.89	75.84	55.67
TDN	67.56	53.91	64.92	77.69	76.85	75.50	72.03

DM = dry matter, OM = organic matter, CP = crude protein, CF = crude fiber, NFE = nitrogen-free extract, TDN = total digestible nutrients.

**Table 4:** Ration formulation and nutritional content of the experimental diet.

Feed ingredient	Ration (%)
Indigofera	7
Elephant grass	40
Gamal	3
Rice bran	10.35
Corn	8.5
Palm kernel meal	18
Sago	12
Minerals	1.15
Total	100
Nutrient content	Value
DM	90.55
OM	90.08
CP	14.36
Ash	9.92
Crude fat	3.06
CF	21.35
NFE	51.45
TDN	65.44

DM = dry matter, OM = organic matter, CP = crude protein, CF = crude fiber, NFE = nitrogen-free extract, TDN = total digestible nutrients.

## RESULTS

### MPS

The effect of the experimental diet on MPS is shown in Table 5.

**Table 5:** Effect of experimental diet on MPS (mg/100 mL).

A factor	B1	B2	B3	B4	Average	SEM
A1	157.37ⁿ ± 3.78	222.59ᵏ ± 2.57	334.47ᵍ ± 3.11	391.85ᶜ ± 2.47	276.57	0.47
A2	172.64ᵐ ± 2.14	252.73ʲ ± 4.34	346.85ᶠ ± 3.11	396.39ᶜ ± 4.68	292.15	
A3	178.83ᵐ ± 1.23	283.28ⁱ ± 1.89	368.73ᵉ ± 3.78	405.47ᵇ ± 3.71	309.08	
A4	188.33ˡ ± 2.57	303.92ʰ ± 6.19	379.47ᵈ ± 2.47	418.27ᵃ ± 3.78	322.50	
Average	174.2936	265.6305	357.3802	402.9971		

*PO* = *Piper ornatum*, LFO = lemuru fish oil, MPS = microbial protein synthesis, SEM = standard error of the mean, A1 = 0% *PO*, A2 = 3% *PO*, A3 = 6% *PO*, A4 = 9% PO, B1 = 0% LFO, B2 = 1.5% LFO, B3 = 3% LFO, B4 = 4.5% LFO. Means with different superscripts in the interaction indicate significant differences (p < 0.05). Means with different superscripts within each column differ significantly (p < 0.05).

The average MPS values ranged from 157.37 to 418.27 mg/100 mL. The combination of *PO* and LFO supplementation resulted in higher MPS than the control. The highest value was observed in treatment A4B4 (9% *PO* + 4.5% LFO) at 418.27 mg/100 mL, whereas the lowest value was found in the control group A1B1 (without *PO* and LFO) at 157.37 mg/100 mL. In general, increasing levels of feed additives were associated with higher MPS values.

### Protozoa population

The effect of the experimental diet on the protozoa population is shown in Table 6.

**Table 6:** Effect of experimental diet on protozoa population (log₁₀ cells/mL rumen fluid).

A factor	B1	B2	B3	B4	Average	SEM
A1	4.19ᶜ ± 0.08	4.24ᵃ ± 0.14	4.25ᵃ ± 0.12	4.17ᵈ ± 0.10	4.21	0.02
A2	4.03ⁱ ± 0.10	4.09ʰ ± 0.31	4.08ʰ ± 0.11	3.95ᵏ ± 0.09	4.04	
A3	4.21ᵇ ± 0.01	4.15ᵉ ± 0.11	3.98ʲ ± 0.12	4.03ⁱ ± 0.26	4.09	
A4	3.98ʲ ± 0.04	4.13ᶠ ± 0.11	4.11ᵍ ± 0.07	3.97ʲ ± 0.25	4.05	
Average	4.10	4.15	4.10	4.02		

*PO* = *Piper ornatum*, LFO = lemuru fish oil, SEM = standard error of the mean, A1 = 0% *PO*, A2 = 3% *PO*, A3 = 6% *PO*, A4 = 9% *PO*, B1 = 0% LFO, B2 = 1.5% LFO, B3 = 3% LFO, B4 = 4.5% LFO. Means with different superscripts in the interaction indicate significant differences (p < 0.05). Means with different superscripts within each column differ significantly (p < 0.05).

The protozoa population obtained in this study ranged from 3.95 to 4.25 × log_10_ cells/mL rumen fluid. The interaction between *PO* and LFO supplementation had a significant effect (p < 0.05) on protozoa values. The highest protozoa population was observed in treatment A1B3 (0% *PO* + 3% LFO) at 4.25 × log_10_ cells/mL rumen fluid, which was not significantly different from A1B2 (0% *PO* + 1.5% LFO) at 4.24 × log_10_ cells/mL rumen fluid, whereas the lowest protozoa population was found in treatment A2B4 (3% *PO* + 4.5% LFO) at 3.95 × log_10_ cells/mL rumen fluid.

### Partial VFA production

The effect of the experimental diet on partial VFA production is shown in Table 7. The production of acetate was significantly influenced by the interaction between *PO* and LFO. The highest acetate concentration was observed in treatment A1B4 (0% *PO* + 4.5% LFO), reaching 78.92 mM, while the lowest was observed in A2B1 (3% *PO* + 0% LFO), with 29.01 mM. In general, the addition of LFO tended to increase acetate production at lower PO levels, but a higher inclusion of *PO* (A4, 9%) showed a more variable response, with acetate values ranging from 49.49 to 74.68 mM. This indicates that the effect of *PO* on acetate production was dose-dependent and strongly modulated by the level of LFO supplementation. For propionate production, a significant interaction was also observed. The highest propionate concentration was observed in treatment A4B3 (9% *PO* + 3% LFO), at 49.10 mM, followed closely by A1B4 (0% *PO* + 4.5% LFO), at 49.04 mM. Conversely, the lowest value was found in treatment A1B1 (0% *PO* + 0% LFO), with only 16.94 mM. Across treatments, the inclusion of LFO generally enhanced propionate formation, particularly at moderate supplementation levels (3–4.5%).

**Table 7:** Effect of experimental diet on partial volatile fatty acid production (mM).

VFA	A factor	B1	B2	B3	B4	Average	SEM
Acetate	A1	34.69ʰ ± 0.44	50.79ᵉᶠᵍ ± 0.02	58.69ᵈᵉ ± 0.03	78.92ᵃ ± 0.07	55.77	0.01
	A2	29.01ʰ ± 0.03	60.52ᶜᵈ ± 0.02	49.31ᶠᵍ ± 0.02	67.42ᵇᶜ ± 0.02	51.57	
	A3	59.57ᶜᵈ ± 0.19	45.94ᵍ ± 0.02	58.87ᵈᵉ ± 0.02	68.00ᵇᶜ ± 0.01	58.10	
	A4	49.49ᶠᵍ ± 0.01	54.40ᵈᵉᶠᵍ ± 0.01	74.68ᵃᵇ ± 0.08	55.05ᵈᵉᶠ ± 0.06	58.41	
	Average	43.19	52.91	60.39	67.35		
Propionate	A1	16.94ᵍ ± 0.10	47.79ᵃᵇ ± 0.03	38.28ᵈ ± 0.04	49.04ᵃ ± 0.07	38.01	0.01
	A2	29.55ᶠ ± 0.04	29.62ᶠ ± 0.04	34.15ᵉ ± 0.03	48.13ᵃᵇ ± 0.01	35.36	
	A3	44.10ᵇᶜ ± 0.05	33.49ᵉ ± 0.06	45.07ᵃᵇ ± 0.03	40.26ᶜᵈ ± 0.11	40.73	
	A4	17.69ᵍ ± 0.13	36.99ᵈᵉ ± 0.09	49.10ᵃ ± 0.01	40.45ᶜᵈ ± 0.08	36.06	
	Average	27.07	36.97	41.65	37.54		
Iso-butyrate	A1	1.25ʰ ± 0.02	3.61ᵃ ± 0.01	2.60ᶜ ± 0.04	3.34ᵃᵇ ± 0.03	2.70	0.01
	A2	1.92ᶠ ± 0.03	2.17ᵉᶠ ± 0.01	2.54ᶜᵈ ± 0.02	3.20ᵇ ± 0.02	2.46	
	A3	2.78ᶜ ± 0.02	2.74ᶜ ± 0.03	3.32ᵃᵇ ± 0.06	2.11ᵉᶠ ± 0.01	2.74	
	A4	1.59ᵍ ± 0.02	2.52ᶜᵈ ± 0.01	2.25ᵈᵉ ± 0.03	2.02ᵉᶠ ± 0.03	2.10	
	Average	1.88	2.76	2.67	2.66		
n-Butyrate	A1	4.18ʰ ± 0.03	12.29ᵃᵇ ± 0.02	9.21ᵉᶠ ± 0.08	13.33ᵃ ± 0.05	9.75	0.01
	A2	7.20ᵍ ± 0.02	7.24ᵍ ± 0.04	8.68ᵉ ± 0.02	11.67ᵇᶜ ± 0.03	8.70	
	A3	11.82ᵇᶜ ± 0.03	8.63ᵉ ± 0.02	11.61ᵇᶜ ± 0.03	9.80ᵈᵉ ± 0.02	10.47	
	A4	4.28ʰ ± 0.02	9.74ᵈᵉ ± 0.05	10.84ᶜᵈ ± 0.06	9.22ᵉᶠ ± 0.04	8.52	
	Average	6.87	9.47	10.08	11.00		
Iso-valerate	A1	0.95ᶠ ± 0.01	3.32ᵃ ± 0.03	2.22ᵉ ± 0.03	3.19ᵃᵇ ± 0.03	2.42	0.01
	A2	1.87ᵉ ± 0.02	2.12ᵉ ± 0.01	2.13ᵉ ± 0.03	3.21ᵃᵇ ± 0.01	2.33	
	A3	3.20ᵃᵇ ± 0.02	2.39ᵈᵉ ± 0.02	2.83ᵇᶜ ± 0.02	2.22ᵉ ± 0.09	2.66	
	A4	1.25ᶠ ± 0.02	2.72ᶜᵈ ± 0.04	2.24ᵉ ± 0.03	2.11ᵉ ± 0.07	2.07	
	Average	1.81	2.63	2.35	2.68		
n-Valerate	A1	0.46ᵍ ± 0.01	1.50ᵃ ± 0.01	1.11ᵈᵉ ± 0.02	1.50ᵃ ± 0.04	1.14	0.01
	A2	0.93ᶠ ± 0.01	0.92ᶠ ± 0.02	1.02ᵉᶠ ± 0.02	1.51ᵃ ± 0.02	1.10	
	A3	1.39ᵃᵇ ± 0.02	1.08ᵈᵉ ± 0.04	1.38ᵃᵇ ± 0.06	1.14ᵈᵉ ± 0.02	1.25	
	A4	0.54ᵍ ± 0.02	1.22ᶜᵈ ± 0.01	1.29ᵇᶜ ± 0.04	1.06ᵉᶠ ± 0.03	1.03	
	Average	0.83	1.18	1.20	1.30		

*PO* = *Piper ornatum*, LFO = lemuru fish oil, VFA = volatile fatty acids, SEM = standard error of the mean, A1 = 0% *PO*, A2 = 3% *PO*, A3 = 6% *PO*, A4 = 9% *PO*, B1 = 0% LFO, B2 = 1.5% LFO, B3 = 3% LFO, B4 = 4.5% LFO. Means with different superscripts in the interaction indicate significant differences (p < 0.05). Means with different superscripts within each column differ significantly (p < 0.05).

The factorial treatments of *PO* and LFO significantly affected the partial production of minor VFA, including iso-butyrate, n-butyrate, iso-valerate, and n-valerate (p < 0.05). For iso-butyrate, the highest concentration was observed in A1B2 (3.61 mM), while the lowest occurred in A1B1 (1.25 mM). Overall, increasing LFO supplementation tended to elevate iso-butyrate production, particularly at moderate inclusion levels, although responses were not strictly linear across combinations. Regarding n-butyrate, a marked increase was observed with high LFO supplementation. The highest production was observed in A1B4 (13.33 mM) and A1B2 (12.29 mM), whereas the lowest was in A1B1 (4.18 mM). The average response across factor B showed that n-butyrate increased progressively with higher LFO levels, ranging from 6.87 mM (B1) to 11.00 mM (B4).

In the case of iso-valerate, the combination A1B2 yielded the highest value (3.32 mM), followed by A2B4 (3.21 mM) and A1B4 (3.19 mM). Conversely, the lowest concentration was recorded at A1B1 (0.95 mM). Mean values indicated that the inclusion of LFO consistently elevated iso-valerate compared with the control (1.81 mM vs. 2.63–2.68 mM). For n-valerate, the peak concentration was observed in A1B2 and A2B4 (both 1.50 mM), while the lowest concentration was observed in A1B1 (0.46 mM).

In summary, iso-butyrate, iso-valerate, and n-valerate production responded positively to the presence of LFO, particularly at moderate to high levels, whereas n-butyrate showed a clear dose-dependent increase with LFO supplementation. Interaction effects with *PO* were evident, but LFO consistently emerged as the dominant factor influencing production of branched- and straight-chain VFA.

### Total VFA

The effect of the experimental diet on total VFA is shown in Table 8.

**Table 8:** Effect of experimental diet on total volatile fatty acid (mM).

A factor	B1	B2	B3	B4	Average	SEM
A1	58.46ʲ ± 0.54	119.29ᵈᵉ ± 0.06	112.10ᵈᵉᶠ ± 0.17	149.32ᵃ ± 0.04	109.79	0.02
A2	70.20ⁱ ± 0.08	102.56ᶠᵍ ± 0.14	97.81ᶠᵍ ± 0.07	135.12ᵇᶜ ± 0.02	101.42	
A3	122.86ᶜᵈ ± 0.25	94.25ᵍʰ ± 0.10	123.06ᶜᵈ ± 0.08	123.51ᶜᵈ ± 0.23	115.92	
A4	74.83ⁱ ± 0.14	107.58ᵉᶠᵍ ± 0.21	140.38ᵃᵇ ± 0.18	109.89ᵈᵉᶠ ± 0.33	108.17	
Average	81.58	105.92	118.33	129.46		

*PO* = *Piper ornatum*, LFO = lemuru fish oil, VFA = volatile fatty acids, SEM = standard error of the mean, A1 = 0% *PO*, A2 = 3% *PO*, A3 = 6% *PO*, A4 = 9% *PO*, B1 = 0% LFO, B2 = 1.5% LFO, B3 = 3% LFO, B4 = 4.5% LFO. Means with different superscripts in the interaction indicate significant differences (p < 0.05). Means with different superscripts within each column differ significantly (p < 0.05).

The total VFA concentration was significantly influenced by the interaction between *PO* and LFO supplementation (p < 0.05). The values ranged from 58.46 to 149.32 mM. The lowest total VFA was observed in the control treatment without *PO* and LFO (A1B1 = 58.46 mM), while the highest concentration was recorded in A1B4 (0% *PO* + 4.5% LFO), reaching 149.32 mM.

From factor LFO, a consistent increase in total VFA was observed with higher supplementation levels, with mean values of 81.58, 105.92, 118.33, and 129.46 mM for B1, B2, B3, and B4, respectively. This indicates that LFO supplementation linearly enhanced ruminal fermentation, leading to higher VFA production. Meanwhile, supplementation of *PO* alone (factor A) showed a more variable response. At 6% inclusion (A3), total VFA tended to be higher (115.92 mM on average) compared with A2 (3%) and A4 (9%), suggesting a dose-dependent effect where moderate supplementation supported fermentation activity. Notably, the combination of 9% *PO* and 3% LFO (A4B3) also yielded a high VFA value (140.38 mM), comparable to that of the highest treatment. These findings suggest that both *PO* and LFO play complementary roles in modulating microbial fermentation and VFA production.

### pH and NH_3_ concentration

The ruminal pH values presented in Table 9 remained stable across all treatments, ranging from 6.90 to 6.99, and were not significantly affected by *PO* or LFO supplementation (p > 0.05). The consistency of average values (6.92–6.94) confirms that neither additive disrupted rumen buffering capacity or microbial stability.

**Table 9:** Effect of experimental diet on pH and NH₃ concentration.

A factor (PO)	B1 (0%)	B2 (1.5%)	B3 (3%)	B4 (4.5%)	Average	SEM
pH						
A1 (0%)	6.95 ± 0.01	6.92 ± 0.06	6.92 ± 0.04	6.95 ± 0.05	6.94	0.01
A2 (3%)	6.92 ± 0.02	6.93 ± 0.01	6.94 ± 0.07	6.92 ± 0.06	6.93	
A3 (6%)	6.90 ± 0.05	6.92 ± 0.03	6.99 ± 0.04	6.90 ± 0.03	6.93	
A4 (9%)	6.91 ± 0.04	6.98 ± 0.05	6.90 ± 0.08	6.91 ± 0.01	6.93	
Average	6.92	6.94	6.94	6.92		
NH_3_ production						
A1 (0%)	18.30ᵍ ± 0.39	18.53ᶠᵍ ± 0.45	17.28ʰ ± 0.98	22.21ᶜ ± 0.87	19.08	0.05
A2 (3%)	18.47ᵍ ± 0.39	19.27ᶠ ± 0.29	21.11ᵈ ± 0.47	23.63ᵃ ± 0.17	20.62	
A3 (6%)	18.42ᵍ ± 0.35	20.17ᵉ ± 0.26	23.72ᵃ ± 0.22	24.57ᵃ ± 0.10	21.72	
A4 (9%)	18.70ᶠᵍ ± 0.17	20.20ᵉ ± 0.40	21.99ᶜ ± 0.52	23.08ᵇ ± 0.26	20.99	
Average	18.47	19.54	21.02	23.37		

*PO* = *Piper ornatum*, LFO = lemuru fish oil, NH₃ = ammonia, SEM = standard error of the mean, A1 = 0% *PO*, A2 = 3% *PO*, A3 = 6% *PO*, A4 = 9% *PO*, B1 = 0% LFO, B2 = 1.5% LFO, B3 = 3% LFO, B4 = 4.5% LFO. Means with different superscripts in the interaction indicate significant differences (p < 0.05). Means with different superscripts within each column differ significantly (p < 0.05).

In contrast, NH_3_ concentration (Table 9) showed a clear interaction effect (p < 0.05). The lowest value (17.28 mg/100 mL) was observed at A1B3, whereas the highest value (24.57 mg/100 mL) occurred at A3B4. A progressive increase in NH_3_ across B1 (18.47 mg/100 mL) to B4 (23.37 mg/100 mL) indicates that LFO enhanced nitrogen degradation. The additional increase observed with *PO* supplementation, particularly at 6%, suggests improved nitrogen release and microbial utilization. Importantly, all NH_3_ values remained within the optimal range required for MPS.

### Nutrient digestibility

All digestibility values shown in Table 10 clearly demonstrate that PO and LFO significantly improved nutrient utilization (p < 0.05). The highest DMD (62.08%), OMD (66.02%), CPD (64.65%), NDFD (60.27%), ADFD (58.37%), CD (63.56%), and HD (65.57%) were consistently observed in treatment A3B4. Compared with the control (A1B1), these improvements indicate enhanced microbial degradation of nutrients, particularly fiber fractions. The data further show that moderate *PO* inclusion (6%) combined with higher LFO levels (4.5%) produced the most efficient fermentation profile.

**Table 10:** Effect of experimental diet on nutrient digestibility (%).

Parameter	A factor (PO)	B1 (0%)	B2 (1.5%)	B3 (3%)	B4 (4.5%)	Average	SEM
DMD	A1 (0%)	58.45ᶠ ± 0.45	59.55ᵉ ± 0.84	61.55ᵇ ± 0.60	61.24ᵇᶜᵈ ± 0.96	60.20	0.09
DMD	A2 (3%)	60.15ᶜᵈᵉ ± 0.65	60.05ᵈᵉ ± 0.47	61.72ᵇ ± 0.51	61.78ᵇ ± 0.69	60.93	
DMD	A3 (6%)	61.40ᵇ ± 0.52	61.65ᵇ ± 0.58	62.07ᵃᵇ ± 0.55	62.08ᵃ ± 0.27	62.05	
DMD	A4 (9%)	60.92ᵇᶜ ± 0.49	61.22ᵇᶜᵈ ± 0.75	61.30ᵇᶜ ± 0.99	61.97ᵃᵇ ± 0.72	61.35	
DMD	Average	60.23	60.61	61.66	64.01		
OMD	A1 (0%)	59.62ᵈ ± 0.59	62.00ᵇᶜ ± 0.90	61.98ᵇᶜ ± 0.55	63.00ᵇ ± 0.66	61.65	0.12
OMD	A2 (3%)	62.42ᵇ ± 0.93	62.25ᵇᶜ ± 0.80	63.02ᵇ ± 1.21	65.00ᵃ ± 0.88	63.17	
OMD	A3 (6%)	61.87ᵇᶜ ± 0.99	62.27ᵇᶜ ± 0.61	62.98ᵇ ± 0.74	66.02ᵃ ± 0.46	63.29	
OMD	A4 (9%)	60.76ᶜᵈ ± 0.98	62.36ᵇᶜ ± 1.31	62.63ᵇ ± 0.99	62.79ᵇ ± 0.63	62.13	
OMD	Average	61.16	62.22	62.65	64.20		
CPD	A1 (0%)	60.06ᶠ ± 0.19	60.29ᶠ ± 0.18	60.94ᵉᶠ ± 0.94	62.04ᶜᵈᵉ ± 0.68	60.83	0.09
CPD	A2 (3%)	60.43ᶠ ± 0.61	61.73ᵈᵉ ± 0.30	63.08ᵇᶜ ± 0.38	63.50ᵃᵇ ± 0.83	62.19	
CPD	A3 (6%)	60.94ᵉᶠ ± 0.88	62.75ᵇᶜᵈ ± 0.78	63.75ᵃᵇ ± 0.94	64.65ᵃ ± 0.52	63.02	
CPD	A4 (9%)	61.29ᵉᶠ ± 0.54	63.45ᵃᵇ ± 1.12	63.80ᵃᵇ ± 0.27	61.79ᵈᵉ ± 0.88	62.58	
CPD	Average	60.68	62.06	62.89	63.00		
NDFD	A1 (0%)	54.25ʰ ± 0.59	54.46ᵍʰ ± 0.37	56.42ᶜᵈᵉ ± 0.66	56.46ᶜᵈ ± 0.27	55.40	0.07
NDFD	A2 (3%)	55.22ᶠᵍ ± 0.69	56.75ᶜ ± 0.23	58.40ᵇ ± 0.24	59.76ᵃ ± 0.13	57.53	
NDFD	A3 (6%)	55.45ᵉᶠ ± 0.06	55.52ᵈᵉᶠ ± 0.26	57.87ᵇ ± 1.04	60.27ᵃ ± 0.86	57.27	
NDFD	A4 (9%)	58.39ᵇ ± 0.57	55.58ᵈᵉᶠ ± 0.42	56.02ᶜᵈᵉᶠ ± 0.44	57.92ᵇ ± 0.57	56.98	
NDFD	Average	55.83	55.58	57.18	58.60		
ADFD	A1 (0%)	52.77ᵍ ± 0.49	53.63ᵉᶠᵍ ± 0.87	54.33ᶜᵈᵉᶠ ± 1.35	55.43ᶜ ± 0.39	54.04	0.09
ADFD	A2 (3%)	53.05ᶠᵍ ± 0.51	55.26ᶜᵈ ± 1.23	53.45ᵉᶠᵍ ± 0.54	57.70ᵃᵇ ± 0.59	54.87	
ADFD	A3 (6%)	54.26ᶜᵈᵉᶠ ± 0.81	53.16ᶠᵍ ± 0.24	53.92ᵈᵉᶠᵍ ± 0.95	58.37ᵃ ± 0.27	54.93	
ADFD	A4 (9%)	56.91ᵇ ± 1.00	53.69ᵉᶠᵍ ± 0.14	54.83ᶜᵈᵉ ± 0.51	56.89ᵇ ± 0.32	55.58	
ADFD	Average	54.25	53.94	54.13	57.10		
CD	A1 (0%)	57.79ᵉ ± 0.57	59.89ᶜᵈ ± 0.54	59.99ᶜᵈ ± 0.86	60.88ᵇᶜ ± 0.74	59.64	0.08
CD	A2 (3%)	59.05ᵈ ± 0.35	60.85ᵇᶜ ± 0.88	58.81ᵈᵉ ± 0.98	61.52ᵇ ± 0.72	59.99	
CD	A3 (6%)	60.74ᵇᶜ ± 0.09	59.13ᵈ ± 0.32	61.42ᵇ ± 0.49	63.56ᵃ ± 0.45	61.21	
CD	A4 (9%)	60.47ᵇᶜ ± 0.42	59.10ᵈ ± 0.86	61.22ᵇ ± 0.48	59.93ᶜᵈ ± 0.41	60.18	
CD	Average	59.51	59.74	60.36	61.47		
HD	A1 (0%)	59.53ᵍ ± 0.83	60.56ᶠᵍ ± 0.28	61.05ᵉᶠ ± 0.56	62.45ᶜᵈ ± 0.73	60.90	0.08
HD	A2 (3%)	63.11ᵇᶜᵈ ± 0.73	62.97ᵇᶜᵈ ± 0.62	63.36ᵇᶜ ± 0.45	64.90ᵃ ± 0.17	63.59	
HD	A3 (6%)	63.14ᵇᶜᵈ ± 0.83	62.98ᵇᶜᵈ ± 0.29	64.92ᵃ ± 0.50	65.57ᵃ ± 0.45	64.15	
HD	A4 (9%)	63.87ᵇ ± 0.55	61.33ᵉᶠ ± 1.04	62.75ᵇᶜᵈ ± 0.39	62.06ᵈᵉ ± 0.65	62.50	
HD	Average	62.41	61.96	63.02	63.75		

*PO* = *Piper ornatum*, LFO = lemuru fish oil, DMD = dry matter digestibility, OMD = organic matter digestibility, CPD = crude protein digestibility, NDFD = neutral detergent fiber digestibility, ADFD = acid detergent fiber digestibility, CD = cellulose digestibility, HD = hemicellulose digestibility, SEM = standard error of the mean, A1 = 0% PO, A2 = 3% PO, A3 = 6% *PO*, A4 = 9% PO, B1 = 0% LFO, B2 = 1.5% LFO, B3 = 3% LFO, B4 = 4.5% LFO. Means with different superscripts in the interaction indicate significant differences (p < 0.05). Means with different superscripts within each column differ significantly (*p* < 0.05).

### Total gas and methane production

The values presented in Table 11 show that PO and LFO significantly reduced both total gas and methane production (p < 0.05). Total gas production decreased from 57.06 mL/g DM in the control (A1B1) to 35.56 mL/g DM in A3B4. Similarly, methane production decreased from 13.60 mL/g DM (A1B1) to 7.46 mL/g DM (A3B4). These reductions clearly indicate that the combination of PO (6%) and LFO (4.5%) was the most effective treatment for suppressing methanogenesis.

**Table 11:** Effect of experimental diet on total gas and methane production.

Parameter	A factor (PO)	B1 (0%)	B2 (1.5%)	B3 (3%)	B4 (4.5%)	Average	SEM
Total gas production (mL/g DM)	A1 (0%)	57.06ᵃ ± 0.12	48.32ᶠᵍ ± 0.86	46.08ᵍʰ ± 2.19	52.50ᵇᶜ ± 1.55	50.99	0.13
Total gas production (mL/g DM)	A2 (3%)	50.91ᶜᵈ ± 0.90	48.31ᶠᵍ ± 0.63	47.01ᶠᵍʰ ± 1.29	48.74ᶠ ± 1.90	48.74	
Total gas production (mL/g DM)	A3 (6%)	52.69ᵇᶜ ± 0.81	48.92ᵈᶠ ± 0.62	46.32ᵍʰ ± 0.84	35.56ʲ ± 0.45	45.87	
Total gas production (mL/g DM)	A4 (9%)	53.98ᵇ ± 1.19	47.71ᶠᵍʰ ± 0.54	40.66ⁱ ± 0.53	45.74ʰ ± 0.06	47.02	
Total gas production (mL/g DM)	Average	53.66	48.32	45.02	45.64		
Methane production (mL/g DM)	A1 (0%)	13.60ᵃ ± 0.06	12.01ᶜᵈ ± 0.50	11.84ᶜᵈ ± 0.43	10.54ᵉᶠ ± 0.04	12.00	0.07
Methane production (mL/g DM)	A2 (3%)	12.29ᵇᶜ ± 0.05	13.17ᵃᵇ ± 0.44	8.93ᵍʰ ± 0.44	8.93ᵍʰ ± 0.50	10.83	
Methane production (mL/g DM)	A3 (6%)	12.00ᶜᵈ ± 0.49	11.71ᶜᵈ ± 0.66	8.33ʰⁱ ± 0.22	7.46ⁱ ± 1.04	9.88	
Methane production (mL/g DM)	A4 (9%)	11.85ᶜᵈ ± 1.30	10.98ᵈᵉ ± 0.26	9.80ᶠᵍ ± 0.43	10.98ᵈᵉ ± 0.43	10.90	
Methane production (mL/g DM)	Average	12.44	11.97	9.73	9.48		

*PO* = *Piper ornatum*, LFO = lemuru fish oil, DM = dry matter, SEM = standard error of the mean, A1 = 0% *PO*, A2 = 3% *PO*, A3 = 6% *PO*, A4 = 9% *PO*, B1 = 0% LFO, B2 = 1.5% LFO, B3 = 3% LFO, B4 = 4.5% LFO. Means with different superscripts in the interaction indicate significant differences (p < 0.05). Means with different superscripts within each column differ significantly (p < 0.05).

The progressive decline across B1 to B4 confirms that increasing LFO levels reduced hydrogen availability for methane formation, while PO contributed antimicrobial effects on methanogenic populations. The consistent pattern across all values in Table 11 supports a strong synergistic interaction between PO and LFO in reducing ruminal gas emissions.

## DISCUSSION

### MPS

The marked increase in MPS, ranging from 157.37 to 418.27 mg/100 mL (Table 5), highlights the synergistic effects of combining PO and LFO as feed additives. This effect may be attributed to bioactive compounds identified in PO in the present study (Table 1), including phenolics and phenylpropanoids (e.g., cinnamaldehyde, coumarin, (E)-4-methoxycinnamic acid), terpenoids (e.g., curcumene, (E,E)-α-farnesene, caryophyllene oxide, nootkatone), sterols (e.g., sitostenone, fucosterol epoxide), and alkaloid-related compounds (e.g., cinchonidine), which have been reported to exhibit antioxidant and antimicrobial activities. These bioactivities may contribute to the modulation of rumen fermentation pathways. Previous chemical studies of Piper species have identified numerous bioactive compounds, including alkaloids, amides, lignans, terpenes, steroids, chalcones, and flavonoids, that exhibit a wide range of biological activities, including antioxidant, antimicrobial, antifungal, anti-inflammatory, and other therapeutic effects [24, 25].

In addition, the modulation of rumen fermentation observed in the present study may be partly explained by the specific fatty acid composition of LFO (Table 2). The LFO was rich in saturated fatty acids (e.g., palmitic and stearic acids), MUFA (e.g., palmitoleic and oleic acids), and PUFA, including linoleic acid, α-linolenic acid, arachidonic acid, and EPA. The presence of PUFA, particularly EPA, may contribute to a hydrogen sink effect in the rumen, potentially shifting fermentation pathways and supporting MPS. Fatty acid profiling strengthens the mechanistic interpretation of LFO effects in the present study, rather than relying solely on general LFO literature. Nevertheless, the specific roles of individual fatty acids in modulating microbial communities and fermentation end-products warrant further targeted investigation. These bioactive compounds interact with microbial cell membranes, thereby altering microbial viability and enzymatic activity [26, 27]. EOs have antimicrobial activities and are considered safe for human and animal consumption [28]. Besides, meta-analyses have shown that EOs can improve rumen fermentability [29].

Furthermore, high PUFA feed supplements, particularly those rich in omega-3 fatty acids, are known to increase dietary energy density and redirect hydrogen utilization away from methanogenesis toward VFA and MPS. This dual action not only reduces methane emissions but also enhances rumen efficiency and microbial growth [30]. The progressive rise in MPS with increasing levels of both feed additives corroborates these mechanisms, suggesting a better rumen environment that supports microbial proliferation and metabolic activity.

The marked increase in MPS highlights the synergistic effects of combining PO and LFO as feed additives. The highest MPS value (418.27 mg/100 mL) was observed in A4B4 (9% *PO* + 4.5% LFO), indicating a strong stimulatory effect on ruminal microbial activity. However, when overall fermentation performance and multiple response variables were considered, A3B4 (6% *PO* + 4.5% LFO) was identified as the best treatment, as it consistently produced high MPS (405.47 mg/100 mL) while maintaining rumen fermentation characteristics across all parameters.

### Protozoa population

The inclusion of *PO* at higher levels (6–9%) generally suppressed protozoal numbers (Table 6), with values ranging from 4.04 to 4.21 log_10_ cells/mL of rumen fluid, indicating the potential antiprotozoal effect of bioactive compounds in *PO*. Meanwhile, supplementation with higher levels of LFO (4.5%) consistently resulted in lower protozoa counts compared to lower doses (4.02–4.15 log_10_ cells/mL). This suggests that PUFA, particularly omega-3 fatty acids (EPA and DHA) from LFO, could impair protozoal cell membranes and reduce their viability.

Overall, although the range of protozoa population values was relatively narrow (3.95–4.25 log_10_ cells/mL rumen fluid), the interaction between *PO* and LFO supplementation suggests a potential combined effect of plant polyphenols and PUFA on rumen protozoa dynamics. However, the magnitude of change was modest, and further mechanistic evidence would be needed to confirm true synergism. In addition to indirect effects mediated by protozoa-associated methanogens, phytogenic compounds may exert direct inhibitory effects on methanogenic archaea, while PUFA from LFO may reduce hydrogen availability for methanogenesis via biohydrogenation.

Meta-analysis has shown that plant-derived EOs, particularly those containing phenolic compounds, exhibit antimicrobial activities that disrupt protozoal cell membranes, leading to significant reductions in protozoal counts [31]. The flavonoids, tannins, and EOs present in *PO* may have exerted similar effects, inhibiting protozoal proliferation by compromising membrane integrity or metabolic function. Moreover, the high PUFA content of LFO (rich in EPA and DHA) likely contributed to reducing protozoal populations by destabilizing protozoal cell membranes. MUFA and PUFA have been reported to insert into microbial lipid bilayers, disrupting membrane fluidity and function, a mechanism that diminishes protozoal viability [32].

In addition, a previous study reported that methanogens belonging to Euryarchaeota showed very low relative abundance and decreased at all levels of EO inclusion [33]. This indicates that the decrease in gas production is due not only to the protozoan population but also to the abundance of archaea. Bacterial population responses were not comprehensively quantified in the present study, and no molecular characterization of the rumen microbiome was performed. Consequently, claims of microbial synergy are based on functional fermentation outcomes rather than direct evidence of shifts in microbial community structure. Future studies employing microbial profiling are required to confirm the specific microbial mechanisms underlying methane mitigation.

### VFA production

The observed modulation of VFA profiles (Tables 7 and 8), particularly the increase in acetate in some treatments (e.g., A1B4) and in propionate in others (notably A4B3), highlights the complex, interaction-dependent effects of combining *PO* and LFO. LFO, rich in long-chain omega-3 PUFAs such as EPA and DHA, is known to alter rumen fermentation by acting as a hydrogen sink and modifying microbial pathways. *In vitro* studies utilizing ruminal batch cultures reported that EPA and DHA supplementation reduced the acetate:propionate ratio while impeding specific hydrogenation steps, demonstrating that these fatty acids can redirect hydrogen flow and promote propionate formation without compromising fermentation [34].

Furthermore, research comparing lipid sources has found that LFO and other marine-derived oils can shift fermentation toward more gluconeogenic VFA, such as propionate, while reducing methane production. *In vitro* trials demonstrated that LFO reduced gas and methane production while altering methanogen and bacterial abundance, thereby influencing VFA proportions even though total VFA remained stable [35]. Similarly, studies using marine oils in continuous fermenter systems confirmed shifts toward propionate along with altered biohydrogenation profiles and reduced methane [36].

The observed enhancement in n-butyrate production with increasing LFO levels suggests that high PUFA inclusion can promote butyric pathways. In a study by Li *et al*. [37], oregano EO increased butyrate production, indicating that lipophilic compounds may enrich butyrogenic microbial populations. Although that study focused on EO rather than LFO, the underlying mechanisms of microbial modulation likely overlap.

Furthermore, the significant increase in iso-butyrate, iso-valerate, and n-valerate at moderate LFO levels suggests increased deamination of branched-chain amino acids. Jiang et al. [38] demonstrated that BCVFA improved fiber digestibility and stimulated the growth of fiber-degrading bacteria, such as Fibrobacter and Treponema, in an *in vitro* rumen model.

Total VFA increased markedly with increasing LFO (Table 8), reaching up to 149 mM in A1B4 compared to 58 mM in the control. Previous studies reported that moderate inclusion of LFO enhances fermentation, whereas excessive levels may suppress it [35].

In contrast, PO supplementation alone (6–9%) produced moderate VFA (~116 mM), indicating a regulatory effect. The highest VFA occurred in combined treatments (e.g., A4B3), suggesting synergy between PO and LFO, in which PO mitigates the potential inhibitory effects of LFO while enhancing fermentation [39, 40].

### pH and NH_3_

Despite the significant supplementation of *PO* and LFO, the ruminal pH across all treatments remained remarkably stable between 6.90 and 6.99 (Table 9). This consistency indicates that the additives did not disrupt the ruminal acid–base balance, thereby maintaining optimal conditions for microbial activity. Similar outcomes were reported by [41], where oil supplementation (pomegranate, garlic, or sunflower oils) at 2% DM had no adverse effect on pH under *in vitro* conditions, reinforcing the idea that moderate levels of unsaturated oils or phytochemicals can be used without impairing fermentative stability.

Lipid supplementation, particularly unsaturated oils from LFO, can alter rumen microbial dynamics without necessarily disturbing ruminal pH. In line with a previous study, Darabighane *et al*. [42] reported that a mixture of sunflower oil and LFO modified nutrient intake and microbial ecology while maintaining ruminal pH within the normal physiological range. This agrees with the present findings (Table 9), where both *PO* and LFO supplementation maintained pH between 6.90 and 6.99. The consistency across studies reinforces the notion that moderate inclusion of PUFA and phytochemical-rich additives does not compromise the acid–base equilibrium of the rumen, even though they may shift fermentation pathways or microbial activity patterns.

Conversely, NH_3_ concentration showed a clear dose-dependent increase with increasing LFO and *PO* levels, peaking at 24.57 mg/100 mL in A3B4 (Table 9). This suggests enhanced proteolysis and deamination processes, likely due to microbial activity stimulated by energy-rich PUFA and possible enzymatic activation by polyphenolic compounds. These NH_3_ values remained within a physiologically acceptable range for supporting MPS. A previous study [43] observed similar trends, in which the inclusion of herbal plants (such as Moringa oleifera and Curcuma longa) significantly increased NH_3_ levels, with values ranging from 13.2 to 17.9 mg/100 mL while maintaining ruminal fermentative balance. This is in line with Vera *et al*. [44], who reported that Pinus radiata bark, which is rich in phenolic compounds, also influenced NH_3_ dynamics. Additionally, the increase in MPS is consistent with NH_3_ production in this study, as sufficient NH_3_ availability facilitates the development of rumen microorganisms.

### Nutrient digestibility

The enhancement of DMD and OMD, particularly pronounced in A3B4 (Table 10), suggests a synergistic benefit from the combination of *PO* and LFO. A related *in vitro* study found that combining LFO with a condensed tannin source (grape seed extract) improved true digestibility, total VFA production, and NH_3_ concentration compared with LFO alone or tannin alone [45]. This supports the hypothesis that LFO provides fermentable energy and hydrogen sinks, while tannin-like compounds modulate microbial proteolysis, together enhancing overall fermentation and digestibility.

Furthermore, the increase in DMD and OMD in A3B4 can be explained by the combined effects of *PO* bioactives and the energy contribution of LFO. Phenolic and flavonoid compounds from *PO* may enhance rumen microflora, thereby improving fermentation efficiency, while LFO provides an accessible lipid source that can be utilized as a microbial energy source. This is consistent with a previous study reporting that secondary plant metabolites, including phenolics, flavonoids, saponins, tannins, and EOs, contribute to livestock productivity and health while also helping control nutritional stress, such as acidosis [46].

CPD notably increased under moderate *PO* (6–9%) combined with LFO, indicating a potential protective effect against rumen proteolysis. This aligns with the findings of Khejornsart *et al*. [47], who reported that supplementation with polyphenol-rich plant material did not impair nutrient utilization or MPS and, in some cases, improved nitrogen capture despite compounds typically considered antinutritional. The moderate level of *PO* inclusion in the present study may have optimally balanced antimicrobial activity with preservation of microbial protein flow.

By comparison, studies on dairy cow rations have shown that synchronization between protein and non-fiber carbohydrates improves DM and OM digestibility, as well as MPS production, demonstrating that balanced fermentation substrates are crucial for maximizing digestive efficiency [48–51].

Fiber digestibility parameters (NDFD, ADFD, CD, and HD) were highest in A2B4 and A3B4 (Table 10). A study in beef cattle that applied tannin-rich plant supplements showed no negative effects on nutrient digestibility, reinforcing the idea that certain levels of phytogenic compounds can enhance fermentation without harming fibrolytic bacteria [47]. LFO may also improve fiber-utilizing microbial populations by enhancing microbial energetic efficiency. The combined effect likely created an optimized rumen environment for fiber degradation, although *PO* (9%) began to decline slightly, suggesting a threshold beyond which phytochemicals may exert inhibitory effects. Crude fiber also influences digestibility, as previous studies have reported that higher crude fiber content is associated with reduced rumen digestibility [50, 52, 53].

### Total gas and methane production

The addition of LFO markedly decreased both total gas and methane production under *in vitro* rumen fermentation conditions, particularly when combined with 6% PO (A3B4) (Table 11). This observation aligns with previous findings [54], which demonstrated that LFO reduced methane emissions by up to 31% when included at 7.5% in a cellulolytic fermentation system. The mechanism is likely related to PUFA’s role as a hydrogen sink, diverting hydrogen away from methanogenesis toward alternative pathways, thereby reducing methane formation without adversely affecting cellulolytic enzyme activity.

Moreover, LFO alone has exhibited substantial antimethanogenic activity in rumen-like systems. Unlike standalone LFO studies showing 17–31% methane reduction with variable effects on fermentation, the combination of *PO* and LFO in the present study achieved approximately 45% methane reduction (from 13.60 to 7.46 mL/g DM; Table 11) while maintaining or improving digestibility. Similarly, a previous study reported methane reductions of 15–20% with lipid supplementation. In comparative *in vitro* studies using dairy cow ruminal inoculum, LFO was more effective than coconut oil at inhibiting methanogens, while total VFA production remained stable [35]. This supports the present findings, in which increasing LFO levels reduced methane output from 12.44 to 9.48 mL/g DM on average (Table 11), demonstrating LFO’s ability to modulate fermentation toward reduced methane production without compromising fermentative efficiency. Similarly, Zain *et al*. [55] reported that the combination of 3% corn oil and 5% chitosan produced a strong synergistic effect in suppressing methane emissions while maintaining overall fermentation.

Furthermore, the modulatory effect of *PO* bioactives on rumen archaeal and bacterial communities may also contribute to methane mitigation beyond simple hydrogen diversion. Meta-analysis data indicate that EOs significantly reduce methane emissions by suppressing protozoa and methanogenic archaea, altering VFA profiles, and reducing NH_3_ [29, 56]. In the present study, flavonoids and phenolic compounds in *PO* likely exert similar selective pressure on methanogens and protozoa, thereby reducing interspecies hydrogen transfer. Flavonoids can disrupt microbial membrane integrity and metabolic activity, thereby reducing gas and methane production without impairing degradability [56].

In addition, PUFA from LFO inhibits biohydrogenation, further contributing to methane reduction. *In vitro* work by Amanullah [39] demonstrated that essential fatty acids constrained biohydrogenation of long-chain PUFA, resulting in lower saturated fatty acid formation and maintaining higher unsaturated intermediates. This reflects a redirection of hydrogen flux away from methanogenesis. In the present study, the combination of *PO* and LFO likely created a dual mechanism, where *PO* modulated microbial communities and LFO redirected hydrogen utilization, together producing a rumen environment that reduced methane while maintaining fermentation efficiency.

The term “optimal” in this study refers to the treatment that achieved the most favorable overall balance among key response variables, rather than the highest value of a single parameter. Specifically, optimality was defined as the combination that maximized methane reduction while maintaining or improving OMD and total VFA and reducing protozoa population. Although individual parameters reached maximum values in different treatments, A3B4 provided the best overall balance and was therefore considered optimal.

This study was conducted under *in vitro* conditions; therefore, rumen outflow dynamics, host–microbe interactions, and microbial adaptation were not evaluated. The additive levels tested may not directly translate to practical in vivo feeding conditions, and the stability and bioactivity of LFO could be influenced by oxidative degradation during handling and incubation. Consequently, in vivo validation is required to confirm the magnitude and persistence of the observed methane mitigation and fermentation responses.

## CONCLUSION

The present *in vitro* study demonstrated that the combined supplementation of *PO* and LFO markedly influenced rumen fermentation characteristics, MPS, nutrient digestibility, and methane mitigation. The results clearly showed that MPS increased substantially from 157.37 to 418.27 mg/100 mL, indicating enhanced microbial growth under combined treatments. Protozoa population exhibited a declining trend (3.95–4.25 log_10_ cells/mL), suggesting suppression of protozoa-associated methanogens. VFA production was significantly modulated, with total VFA increasing to 149.32 mM, compared with 58.46 mM in the control, reflecting improved fermentative efficiency. Ruminal pH remained stable between 6.90 and 6.99, confirming that *PO* and LFO did not disrupt rumen buffering capacity, while NH_3_ concentration increased up to 24.57 mg/100 mL, supporting enhanced microbial activity and MPS. Nutrient digestibility parameters, including DMD, OMD, CPD, NDFD, ADFD, CD, and HD, were significantly improved, with the highest values consistently observed under combined treatments. Moreover, total gas and methane production were markedly reduced, with methane decreasing from 13.60 to 7.46 mL/g DM, indicating a strong antimethanogenic effect.

From a practical perspective, integrating *PO* as a phytogenic additive with LFO as a PUFA-rich lipid source offers a promising nutritional strategy to improve rumen efficiency and reduce enteric methane emissions. The dual mechanism involving antimicrobial activity of plant bioactives and hydrogen redirection by PUFA provides an effective approach to enhance MPS while mitigating environmental impacts. This strategy is particularly relevant for sustainable ruminant production systems where reducing greenhouse gas emissions without compromising productivity is essential.

The strength of this study lies in its factorial experimental design, which enabled a clear evaluation of the interaction effects between *PO* and LFO across multiple inclusion levels. Additionally, the comprehensive assessment of fermentation parameters, including MPS, protozoa population, VFA profiles, NH_3_ concentration, nutrient digestibility, and methane production, provides a holistic understanding of rumen responses. The inclusion of detailed chemical characterization of *PO* and fatty acid profiling of LFO further strengthens the mechanistic interpretation of the results.

However, several limitations should be acknowledged. The study was conducted under *in vitro* conditions, which do not fully replicate the complexity of the rumen environment under in vivo conditions, including host–microbe interactions, rumen turnover, and long-term microbial adaptation. Furthermore, microbial populations were not directly quantified using molecular techniques, limiting the ability to confirm specific shifts in microbial community structure. The stability of LFO and potential oxidative degradation during incubation may also influence the observed responses.

Future research should focus on validating these findings under in vivo conditions, including long-term feeding trials to evaluate animal performance, feed efficiency, and product quality. Detailed microbial profiling using advanced molecular approaches is required to elucidate the mechanisms underlying the observed changes in fermentation and methane production. Additionally, the optimization of inclusion levels across different dietary systems and the evaluation of economic feasibility should be explored to facilitate practical application.

In conclusion, the combined supplementation of *PO* and LFO provides an effective strategy to enhance rumen fermentation, improve nutrient digestibility, increase MPS, and significantly reduce methane production. Among the treatments, A3B4 (6% *PO* + 4.5% LFO) achieved the most favorable balance across all response variables, making it the optimal treatment under *in vitro* conditions. These findings highlight the potential of integrating phytogenic additives with PUFA-rich lipid sources as a sustainable approach for improving ruminant productivity while mitigating environmental impacts.

## DATA AVAILABILITY

The supplementary data can be made available from the corresponding author upon request.

## AUTHORS’ CONTRIBUTIONS

MZ, DD, UHT, and YY: Conceptualization, study design, supervision, and writing – original draft. EMP and RP: Data interpretation, critical revision of the manuscript for important intellectual content, and writing – review and editing. GY, ZI, RRS, LSS, and BVU: Laboratory analysis and writing the manuscript. MZ, GY, and ZI: Data analysis. All authors have read and approved the final version of the manuscript.
